# A Zebrafish Model of *Mycobacterium leprae* Granulomatous Infection

**DOI:** 10.1093/infdis/jix329

**Published:** 2017-07-18

**Authors:** Cressida A Madigan, James Cameron, Lalita Ramakrishnan

**Affiliations:** 1 Department of Microbiology,; 2 Department of Immunology, and; 3 Department of Medicine, University of Washington, Seattle; and; 4 Molecular Immunity Unit, Department of Medicine, University of Cambridge, MRC Laboratory of Molecular Biology, United Kingdom

**Keywords:** zebrafish, mycobacteria, leprosy, granuloma

## Abstract

Understanding the pathogenesis of leprosy granulomas has been hindered by a paucity of tractable experimental animal models. *Mycobacterium leprae*, which causes leprosy, grows optimally at approximately 30°C, so we sought to model granulomatous disease in the ectothermic zebrafish. We found that noncaseating granulomas develop rapidly and eventually eradicate infection. *rag1* mutant zebrafish, which lack lymphocytes, also form noncaseating granulomas with similar kinetics, but these control infection more slowly. Our findings establish the zebrafish as a facile, genetically tractable model for leprosy and reveal the interplay between innate and adaptive immune determinants mediating leprosy granuloma formation and function.

## Summary

The study of leprosy is hampered by the paucity of animal models. This study describes the development of the genetically tractable zebrafish to study leprosy pathogenesis, particularly granuloma formation and function.

Few animal models exist for the study of *Mycobacterium leprae* pathogenesis in vivo, largely because the ≥37°C core temperature of traditional rodent models prevents *M. leprae* survival [1]*. M. leprae* is propagated for research use in the athymic mouse footpad [1], where it induces granuloma formation but not the neurological disease typical of human leprosy [2]. Armadillos develop neurological disease and form granulomas in response to *M. leprae*; however, they do not breed in captivity and lack most genetic, molecular, and immunological tools [3]. Cultured macrophages have been used to model early granuloma formation with *M. leprae,* but the scope of this model remains limited [4]. Overall, the host determinants that mediate granuloma formation in leprosy and their role in pathogenesis are incompletely understood.

The zebrafish has become an effective model for studying *Mycobacterium tuberculosis* granulomas by using *Mycobacterium marinum*, the agent of fish tuberculosis and a close genetic relative of the *M. tuberculosis* complex [5]. *M. marinum* infection of adult zebrafish results in organized, multicentric granulomas that become necrotic, similar to those of human tuberculosis [6]. Zebrafish are housed at approximately 30°C, similar to the optimum growth temperature of *M. leprae*; indeed, a more than century-old article reports experimental *M. leprae* infection of several fish species [7]. Therefore, we explored the zebrafish as a leprosy model, with a focus on granuloma development, fate, and function.

## METHODS

Zebrafish husbandry and experiments were conducted at the University of Washington in compliance with guidelines from the National Institutes of Health and were approved by the University of Washington Institutional Animal Care and Use Committee. Four-month-old male zebrafish, either wild-type AB strain or sibling *rag1*^*t26683/t26683*^ mutants and *rag1*^*+/t26683*^ heterozygotes, were infected intraperitoneally (as described elsewhere [6]) with 5 × 10^7^*M. leprae* isolated from mouse footpads; bacteria were tested for viability by radiorespirometry, as described previously [1]. *rag1*^*t26683/t26683*^ and *rag1*^*+/t26683*^ were identified among offspring from a *rag1*^*+/t26683*^ incross by genotyping, using high-resolution melt analysis of amplicons generated with primers GCGCTATGAGATCTGGAGGA and TGCAGTGCATCCAGAGTAGG or primers GCGCTAT GAGATCTGGAGGA and CAGAGTAGGCTGGGTTTCCA on a CFX Connect Thermocycler (BioRad). Animals were observed twice daily and culled by tricaine overdose at each experimental time point or, in the survival experiment, if they appeared moribund. To measure bacterial burden, we used histologic analysis with Fite staining to detect bacilli, which is the typical method for diagnosis of human leprosy [8, 9]. Sections were prepared for histologic analysis as described elsewhere [6]. Briefly, serial sagittal sections were made from formalin-fixed animals and stained by hematoxylin-eosin to visualize host cells and by Fite, a modified acid-fast stain, to visualize *M. leprae* organisms, which are acid-fast bacilli. Sections were examined using bright-field microscopy, and images were collected with a digital photo camera (model DKC-5000; Sony, Tokyo, Japan) and produced using Metamorph software (Molecular Devices Corporation, Sunnyvale, CA). Three fish per group per time point were examined. As a surrogate for bacterial burden per fish, Tissue Studio 4.0 (Definiens) was used to identify the acid-fast bacilli–positive regions in a single sagittal section and measure their cumulative area. Animals were considered to have cleared infection if no acid-fast bacilli were detected in the entire sagittal section. Serial sagittal sections (3–4 per animal) were examined to confirm that there were no significant differences between the sections and that the sections were representative (Supplementary Figure 1*A*). Statistical analyses were performed using Prism (version 5.0a; GraphPad).

## RESULTS

**Figure 1.**
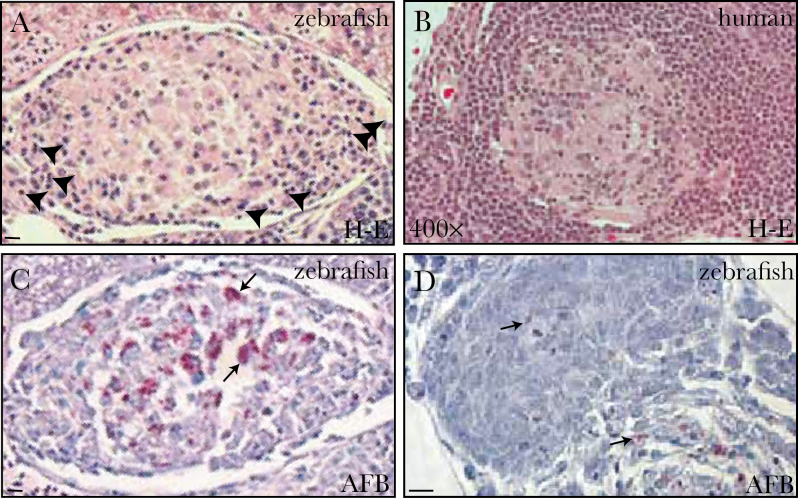
Adult zebrafish are susceptible to *Mycobacterium leprae* infection. *A*, Hematoxylin-eosin (H-E)–stained section of a granuloma in the peritoneal cavity of a wild-type adult zebrafish 7 days after infection with 5 × 10^7^ Thai53 strain *M. leprae*. Arrowheads indicate lymphocyte nuclei. *B*, Granuloma from a skin biopsy specimen from a patient with tuberculoid leprosy. The image is from the archives of the Lauro de Souza Lima Institute. *C*, Serial section of the granuloma in panel *A*, stained for acid-fast bacilli (AFB) to detect *M. leprae*; many bacteria are present (arrows). *D*, AFB-stained granuloma section from the peritoneal cavity of a similarly infected fish, 7 days after infection; few bacteria are present. Arrows indicate bacilli. Bars denote 10 μm.

A total of 5 × 10^7^*M. leprae* were injected into zebrafish, similar to the number of bacteria used to inoculate mouse footpads [1]. Within 7 days after infection with *M. leprae*, zebrafish had formed organized granulomas throughout the body, involving the pancreas, liver, intestine, mesentery, blood vessels, gonad, and adipose tissue (Figure 1A). The granulomas were composed centrally of macrophages that had undergone epithelioid transformation (characterized by a high cytoplasm to nucleus ratio), with scattered lymphocytes (characterized by abundant eosinophilic cytoplasm and indistinct cytoplasmic borders) aggregating at the periphery (Figure 1A). Thus, even from this early stage, they resembled the organized granulomas of human leprosy (Figure 1B). Fite staining revealed that similarly sized granulomas within the same fish contained varying numbers of bacteria, possibly reflecting ongoing bacterial killing (Figure 1C and 1D).

We sought to determine the role of adaptive immunity in the control of leprosy. For tuberculosis, the critical role of adaptive immunity in the control of infection is highlighted by the role of human immunodeficiency virus (HIV) infection in increasing susceptibility to *M. tuberculosis* infection [10]. *rag1* mutant mice lacking mature T and B cells are hypersusceptible to *M. tuberculosis* [5]. Likewise, SCID mice, also lacking mature T and B cells, have increased *M. leprae* burdens in their footpads, which decreases upon administration of T cells to the animals [11]. However, the role of adaptive immunity in the control of human leprosy is unclear. On the one hand, lymphocytes are present in the well-organized granulomas of paucibacillary leprosy, similar to the case with tuberculous granulomas in humans, and an effective cellular response is associated with paucibacillary leprosy [5, 8]. On the other hand, the evidence that HIV infection exacerbates leprosy in humans is scant, with only isolated reports of increased tendency for multibacillary disease, reactions, and relapse [12].

**Figure 2.**
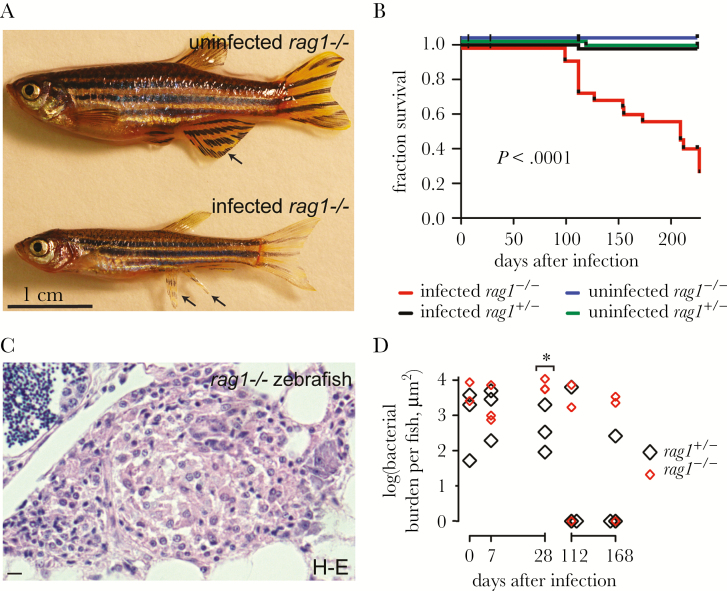
Adaptive immunity contributes to control of *Mycobacterium leprae* infection. *A*, Representative images of sibling uninfected and infected *rag1* mutant animals approximately 100 days after infection; the *M. leprae*–infected animal is smaller than the uninfected animal. Arrows indicate an intact fin in the uninfected animal and a frayed fin in the infected animal. *B*, Kaplan-Meier survival curve of sibling *rag1* heterozygote and mutant zebrafish with or without infection due to *M. leprae* as described in Figure 1A. There were 61 uninfected heterozygotes, 20 infected heterozygotes, 57 uninfected mutants, and 41 infected mutants. *C*, Hematoxylin-eosin (H-E)–stained section of a *rag1* mutant zebrafish granuloma, infected as described in Figure 1A. Bar denotes 10 μm. *D*, Quantification of bacterial burden per fish in *rag1* heterozygotes and mutants. *P = .03, by the Student *t* test, comparing heterozygotes to mutants at each time point. Other comparisons were not significant.

We previously showed that *rag1* mutant zebrafish are more susceptible to *M. marinum*, recapitulating the findings of *rag1* mutant mice infected with *M. tuberculosis* [5, 6]. We asked whether *rag1* mutant zebrafish were also more susceptible to *M. leprae.* We compared them to their heterozygous siblings, which are as resistant as wild-type fish to *M. marinum* [6]. By approximately 60 days after infection, the infected mutants had become runted with frayed fins (Figure 2A) and began to die soon after (Figure 2B). Decreased survival was statistically significant in the infected *rag1* mutants but not the other groups (Figure 2B), and all dying animals manifested similar signs of disease before death (runting, frayed fins, hemorrhaging, and swimming near the tank bottom). Only 3 of 12 infected mutants survived, and these survivors appeared healthy, suggesting that some mutants were able to clear infection.

Simultaneously, in a separate small cohort (3 *rag1* heterozygote and 3 mutant animals per time point), we performed tissue histologic analysis to assess granuloma morphology and bacterial burdens. *rag1* mutants formed organized epithelioid granulomas by 7 days that were similar to those for wild-type fish except that, as expected, they lacked lymphocytes (Figures 2C). Analysis of Fite-stained histologic sections suggested that both heterozygotes and mutants cleared infection over time. At 112 days after infection and 168 days after infection, 2 of 3 *rag1* heterozygotes contained no bacilli, while 1 of 3 *rag1* mutants contained no bacilli (Figure 2D and Supplementary Figure 1*A*).

In the remaining animals, we assessed bacterial burdens at various time points by quantifying Fite-positive bacteria in multiple sections in each animal (Supplementary Figure 1*A*). We found that, in the remaining animals, mutant bacterial burdens were greater than in heterozygotes at 28 days and then declined (Figure 2D). Together, these findings suggest that although adaptive immunity is important in controlling *M. leprae*, it can be controlled by innate immunity alone. Whether these differences reflect differences in bacterial replication, bacterial killing, or both awaits the development of direct assays for bacterial replication in vivo.

A curious feature of *M. leprae* granulomas is that they seldom become necrotic, even when laden with organisms [8]; this is in sharp contrast to human tuberculous granulomas [5]. In the zebrafish too, we found that even multibacillary lesions where individual macrophages were packed with bacteria seldom became necrotic (Supplementary Figure 1*B*). Necrosis was observed in only 2.9% of heterozygote granulomas (1 of 34 granulomas in 12 animals; Supplementary Figure 1*C*). Similarly, only a minority of the *rag1* mutant granulomas (14% [7 of 50] in 12 animals) became necrotic; this difference was not statistically significant.

Finally, human leprosy granulomas are frequently associated with damage to peripheral nerves. We were unable to assess nerve damage in this study, as even an experienced neuropathologist was unable to identify the nerves in these small animals. In a companion study using zebrafish larvae, which are transparent, we have been able to show the association between early macrophage aggregates and nerve injury [13].

## Discussion

This pilot study suggests the promise of the adult zebrafish as a model for studying *M. leprae* granuloma formation and function and the immune pathways that determine host susceptibility to leprosy. Morphologically, most granulomas resemble those of paucibacillary (or tuberculoid) human leprosy, and, like their human counterparts, they are effective in controlling infection [14]. Indeed, the vast majority of humans appear to clear *M. leprae* infection [14], and most zebrafish do as well. As with humans, our data suggest that the ability of zebrafish to clear *M. leprae* infection differs among individuals. This likely reflects varied immune responses in the zebrafish, which, like humans, are outbred (in contrast, mice are inbred). Dr Richard Truman at the National Hansen’s Disease Programs found a similarly high degree of fish-to-fish variability when he used *M. leprae* to infect medaka, another outbred fish species (personal communication, 19 May 2017).

Another intriguing feature of human leprosy is the rarity of granuloma necrosis [8], and this too is preserved in zebrafish. This could be because *M. leprae* has lost determinants present in *M. marinum* and *M. tuberculosis* that promote granuloma macrophage necrosis.

Finally, our work reveals the complexity of the interplay between innate and adaptive immunity in the control of leprosy. In separate work, we developed the larval zebrafish as a leprosy model, and we found that macrophages can aggregate into granulomas and control *M. leprae* to a substantial extent in the sole context of innate immunity [13]. Our findings here, with the *rag1* mutant, reinforce the idea that bona fide epithelioid granulomas form without adaptive immunity [5], yet the full microbicidal capacity of the granuloma macrophages requires stimulation by adaptive immunity. Indeed, we found that lymphocytes begin to arrive in the granuloma by 7 days after infection and that bacterial burdens diverge between *rag1* heterozygotes and mutants by 28 days (Figure 2D). Thereafter, bacterial burdens decreased even in the *rag1* mutant fish, suggesting that innate immune factors can gradually control infection (Figure 2D). The finding that mutants slowly reduce bacterial burdens and occasionally even clear infection suggest that innate immunity alone may be sufficient to control this slowly growing pathogen. The decreased survival of *rag1* mutants in the face of this delayed control may reflect the adverse consequences of chronic infection or be due to cytokine dysregulation in the absence of adaptive immunity. In any case, our zebrafish findings may reflect the lack of an obvious link between exacerbation of leprosy and HIV coinfection [12]. Moreover, given that innate immunity has a role in clearing infection, the development in humans of multibacillary rather than paucibacillary leprosy may well reflect innate immune deficiencies, some of which are beginning to be identified [8, 15]. It is our hope that these can be broadly identified and studied in the zebrafish, using the publicly available libraries of zebrafish mutants that have been generated by chemical mutagenesis and CRISPR (clustered regularly interspaced short palindromic repeats) technologies.

## Supplementary Data

Supplementary materials are available at *The Journal of Infectious Diseases* online. Consisting of data provided by the authors to benefit the reader, the posted materials are not copyedited and are the sole responsibility of the authors, so questions or comments should be addressed to the corresponding author.

## Supplementary Material

Supplementary Figure_S1

Supplementary Figure_Legend

## Notes

***Acknowledgments.*** We thank the National Hansen’s Disease Program, Healthcare Systems Bureau, Health Resources and Services Administration, Department of Health and Human Services (Baton Rouge, LA), with financial support from the National Institute of Allergy and Infectious Diseases (grant IAA-2646), for live *M. leprae*; Christine Cosma, for advice on and technical assistance with zebrafish infections; Paul Edelstein, for discussions, advice on histologic preparations, and statistical analysis, and for manuscript review; Robert Modlin, for advice on histologic analysis and for manuscript review; and Philip Scumpia, for advice on histologic interpretation.

***Financial support.*** This work was supported by the National Institutes of Health (grant T32 AI1007411) and National Research Service Award AI104240 to C. A. M, and MERIT award R37AI054503 and the Director’s Pioneer Award to L. R. C. A. M. is an A. P. Giannini Foundation postdoctoral fellow and L. R. is a Wellcome Trust Principal Research Fellow.

***Potential conflicts of interest.*** All authors: No reported conflicts of interest. All authors have submitted the ICMJE Form for Disclosure of Potential Conflicts of Interest. Conflicts that the editors consider relevant to the content of the manuscript have been disclosed.
