# Supplementary figures and images for: A Zebrafish Model of Mycobacterium leprae Granulomatous Infection
Source: J Infect Dis. 2017 Jul 18;216(6):776–9. doi: 10.1093/infdis/jix329 (PMC5853370; doi:10.1093/infdis/jix329)

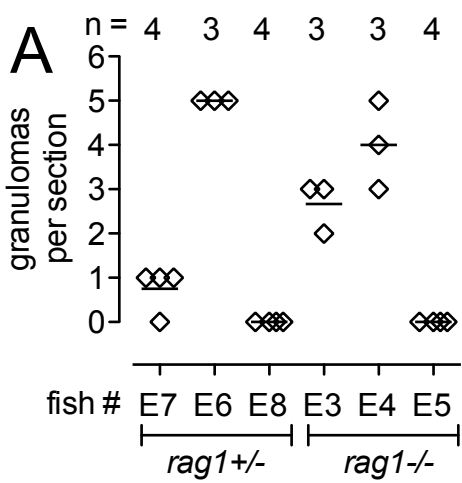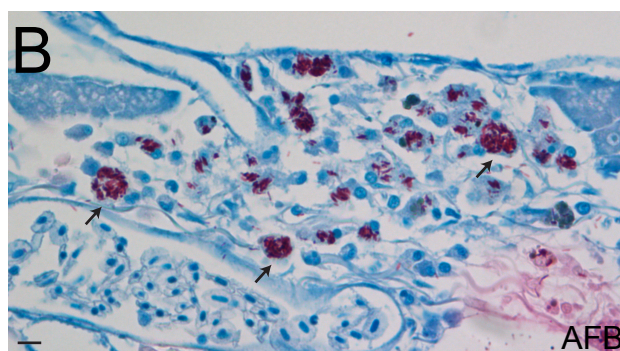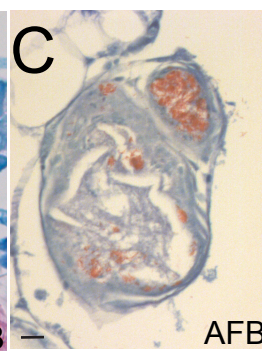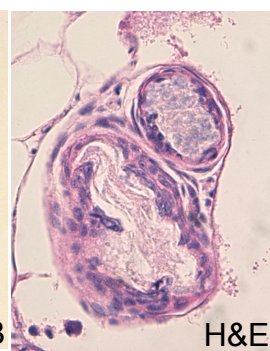

Supplement: Supplementary Figure_S1 [file jix329_suppl_supplementary_figure_s1.pdf]
