# Supplementary material for: A Zebrafish Model of Mycobacterium leprae Granulomatous Infection
Source: J Infect Dis. 2017 Jul 18;216(6):776–9. doi: 10.1093/infdis/jix329 (PMC5853370; doi:10.1093/infdis/jix329)
Supplement: Supplementary Figure_Legend [file jix329_suppl_supplementary_figure_legend.docx]

**Supplementary Figure Legend**

**Supplemental Figure 1.** Detailed analysis of *M. leprae* granulomas. In panel A, multiple AFB-stained sections from infected fish at 112 dpi were scored for number of infected granulomas; n, number of sections scored. In panel B, an AFB-stained section of a non-necrotizing granuloma in a *rag1* heterozygote zebrafish with heavily infected macrophages (arrows). In panel C, AFB and H&E sections of a necrotic granuloma observed in *M. leprae*-infected *rag1* heterozygote fish. 10μm bars.
